# Effect of α-Tocopherol on the Physicochemical Properties of Sturgeon Surimi during Frozen Storage

**DOI:** 10.3390/molecules24040710

**Published:** 2019-02-15

**Authors:** Shuwei Tang, Guangxin Feng, Wenxuan Cui, Ruichang Gao, Fan Bai, Jinlin Wang, Yuanhui Zhao, Mingyong Zeng

**Affiliations:** 1 College of Food Science and Engineering, Ocean University of China, Qingdao 266003, China; T13061427625@163.com (S.T.); fgx15653279039@163.com (G.F.); 2 Penglai Aquatic Product Technology Promotion Department, Yantai 265600, China; zzwxc@126.com; 3 School of Food and Bioengineering, Jiangsu University, Zhenjiang 212013, China; xiyuan2008@ujs.edu.cn; 4 Hangzhou Qiandaohu Sturgeon Technology Co., Ltd., Hangzhou 310000, China; bf@kalugaqueen.com (F.B.); wjl635280708@126.com (J.W.)

**Keywords:** sturgeon surimi, physicochemical properties, α-tocopherol, frozen storage

## Abstract

This study investigated the effects of α-tocopherol (α-TOH) on the physicochemical properties of sturgeon surimi during 16-week storage at −18 °C. An aliquot of 0.1% (*w*/*w*) of α-TOH was added into the surimi and subjected to frozen storage, and 8% of a conventional cryoprotectant (4% sorbitol and 4% sucrose, *w*/*w*) was used as a positive control. Based on total viable count, pH and whiteness, α-TOH exhibited a better protection for frozen sturgeon surimi than cryoprotectant during frozen storage. According to soluble protein content, carbonyl content, total sulfhydryl content, and surface hydrophobicity, α-TOH and cryoprotectant showed the same effects on retarding changes of proteins. The results of breaking force, deformation, gel strength, water-holding capacity and microstructure of sturgeon surimi indicated that the gel properties of frozen sturgeon surimi were retained by α-TOH. Our results suggest that α-TOH is an attractive candidate to maintain the quality of sturgeon surimi during frozen storage.

## 1. Introduction

Surimi is a wet concentrated myofibrillar protein made by mechanical bone removal, successive washing, and freezing of chopped fish. Surimi can be produced from both marine and freshwater fish, including less popular and underused fish species [1]. Sturgeons (*Acipenseridae*) are one of the largest fish species in the world and have high economic value [2]. Recently, sturgeon aquaculture has developed rapidly in many countries. Especially in China, the aquaculture of sturgeon accounts for about 86% of the global sturgeon meat production [3,4]. However, the utilization of sturgeon meat is limited. Sturgeon meat has no bones, and thus the meat loss is reduced due to the absence of filtration steps in the preparation of sturgeon surimi [5]. In addition, sturgeon meat has a high protein content and is rich in many essential amino acids, which are extremely important for human health. Therefore, sturgeon meat may be a good choice for exploitation and utilization as a surimi raw material.

**Figure 1:**
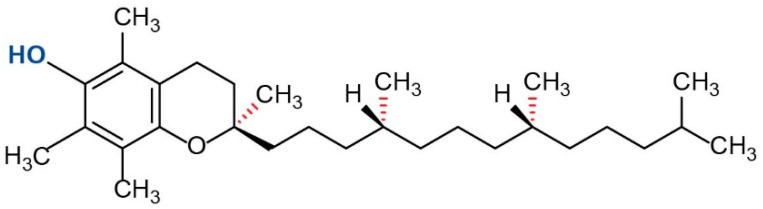
Structure of α-tocopherol.

Surimi is stored and transported in a frozen state. Over the past decade, there has been growing production of frozen surimi because of increasing consumer demand. However, during frozen storage, surimi undergoes denaturation, aggregation and a loss of functional properties [6]. Apart from the ice-crystal-induced destruction of hydrate layers resulting in fish denaturation, protein oxidation also is a major cause for quality losses during frozen storage. It is well known that surimi protein denaturation can be suppressed by cryoprotectants such as low-molecular-weight sugars, polyols, and starch hydrolysates [7]. While effective, they make the surimi too sweet and affect the taste of surimi, so the resulting surimi products are not as desirable to consumers. It seems to be possible to preserve frozen surimi with antioxidants to prevent protein oxidation. α-Tocopherol (α-TOH) is a type of vitamin E, which can reduce free radicals by donating a hydrogen atom from its hydroxyl group. The fully methylated form of α-TOH (Figure 1) is by far the most potent form of vitamin E and it has been approved as a food additive used as an antioxidant in many countries [8]. Compared to other forms of vitamin E, α-TOH is well-absorbed and well-accumulated in humans [9]. α-TOH has been extensively utilized for inhibition of lipid oxidation in meats [10], however, there is limited information regarding its effectiveness for suppression of protein oxidation in surimi. Recently, Wang et al. reported that α-TOH could retard protein oxidation of fish mince during frozen storage [11]. Therefore, it may be possible to use α-TOH for protecting frozen surimi from oxidation-induced protein denaturation during frozen storage.

The present study investigated the effects of α-TOH on the physicochemical properties of frozen sturgeon surimi during frozen storage, including total viable counts, pH, salt-extractable protein, water-extractable protein, protein carbonyls, protein total sulfhydryl, surface hydrophobicity, gel properties, water holding capacity, whiteness and microstructure.

## 2. Results and Discussion

### 2.1. Effects of α-TOH on Total Viable Counts and pH of Sturgeon Surimi during Frozen Storage

**Figure 2:**
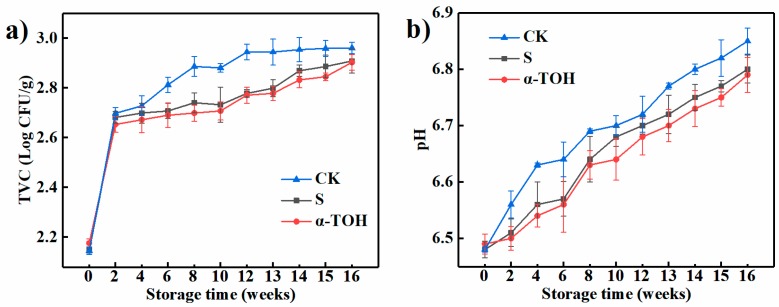
Effects of α-TOH on the total viable count (**a**) and pH value (**b**) of sturgeon surimi during 16-week frozen storage at −18 °C. Bars represent the standard deviation from triplicate determinations (*n* = 3; 3 means 3 replicates) of the sturgeon surimi gels in different temperature treatments.

The total viable counts (TVC) is an important indicator of meat quality during frozen storage and commonly used in the studies on the shelf-life of meat [12]. As shown in Figure 2a, after 16-week frozen storage, TVC values of the control (CK) group, conventional cryoprotectant (S) group and α-TOH group were respectively 2.96 ± 0.029, 2.91 ± 0.049 and 2.90 ± 0.031 log CFU/g. Compared to the TVC values of fresh sturgeon surimi (Week 0), those of CK group increased by 38.04% after 16-week frozen storage, which was significantly (*p* < 0.05) higher than the S (35.51%) and α-TOH groups (33.41%), suggesting that both conventional cryoprotectant and α-TOH inhibited bacterial growth. In addition, TVC values of three groups were lower than 6.0 log CFU/g (the recommended limits) [13], indicating that frozen sturgeon surimi after 16 weeks of frozen storage still had a good quality.

For aquatic products, pH is a common quality indicator [14]. As shown in Figure 2b, the initial pH values of the CK group, S group and α-TOH group were respectively 6.48 ± 0.005, 6.48 ± 0.015 and 6.49 ± 0.018, which was lower than pH (6.9) of bighead carp (*Aristichthys nobilis*) [15]. Generally, the initial pH of aquatic products is affected by species, level of enzyme activity, season, and other factors [16]. After 16 weeks of frozen storage, the pH of the CK group, S group and α-TOH group increased to 6.85 ± 0.023, 6.80 ± 0.025 and 6.79 ± 0.031, respectively, which were close to the neutral value. The increase of pH in surimi may be due to that the proteins of frozen sturgeon surimi underwent degradation with the extension of storage time, resulting in the production of the volatile compounds and ammonia [17]. In addition, the pH of CK group was consistently higher than that of α-TOH group (*p* < 0.05) during the whole frozen storage period, suggesting that α-TOH effectively prevented the protein degradation occurring during frozen storage.

### 2.2. Effects of α-TOH on the Water-Extractable Protein (WEP) and Salt-Extractable Protein (SEP) of Sturgeon Surimi during Frozen Storage

**Figure 3:**
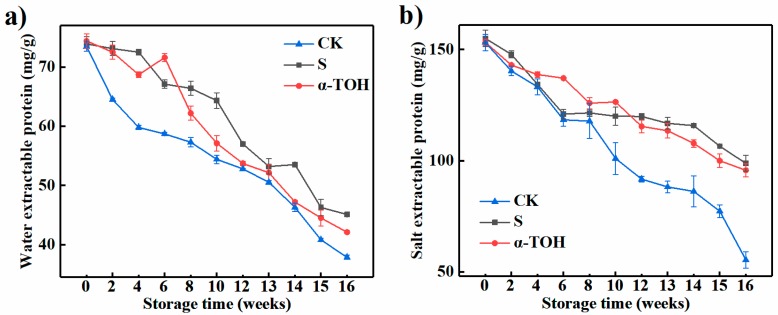
Effects of α-TOH on the water-extractable protein content (**a**) and salt-extractable protein content (**b**) of sturgeon surimi during 16-week frozen storage at −18 °C. Bars represent the standard deviation from triplicate determinations (*n* = 3; 3 means 3 replicates).

Muscle proteins are conveniently characterized by their solubility properties and can be divided into salt-extractable/myofibrillar protein, water-extractable/sarcoplasmic protein and insoluble/muscle matrix protein according to the protein solubility [18]. As presented in Figure 3, with the increase of frozen storage time, the SEP and WEP contents of all groups decreased significantly (*p* < 0.05). In general, the WEP content was lower than the SEP content throughout 16-week storage (*p* < 0.05) because surimi was made from successive washing minced fish, in which sarcoplasmic proteins were removed [19]. According to the report of Kong et al. [20], the change of SEP content was an important parameter to indicate protein denaturation resulting from the formation of hydrophobic bonds or hydrogen, as well as ionic interactions and disulfide bonds.

WEP and SEP of CK group after 16 weeks were reduced by 48.45% and 63.81% compared with that in fresh surimi (Week 0). When cryoprotectants were not added, the rapid decrease in protein content during frozen storage was significant in other fish muscle systems [21]. WEP of S and α-TOH groups after 16-week frozen storage was reduced by 38.97% and 43.41% than that in fresh sturgeon surimi (Week 0). And after 16 weeks, the SEP values in the S and α-TOH groups were respectively 98.80 ± 3.559 and 95.70 ± 2.867 mg/g, indicating conventional cryoprotectant and α-TOH had a good protective effect on sturgeon surimi. In addition, after 16 weeks of frozen storage, there was no significant difference in SEP between α-TOH group and S group. This result indicated that the protective effects of α-TOH on sturgeon surimi was comparable to those of the conventional cryoprotectant, and α-TOH could protect salt-extractable protein from oxidation.

### 2.3. Effects of α-TOH on the Protein Carbonyls, Protein Total Sulfhydryl and Surface Hydrophobicity of Sturgeon Surimi during Frozen Storage

**Figure 4:**
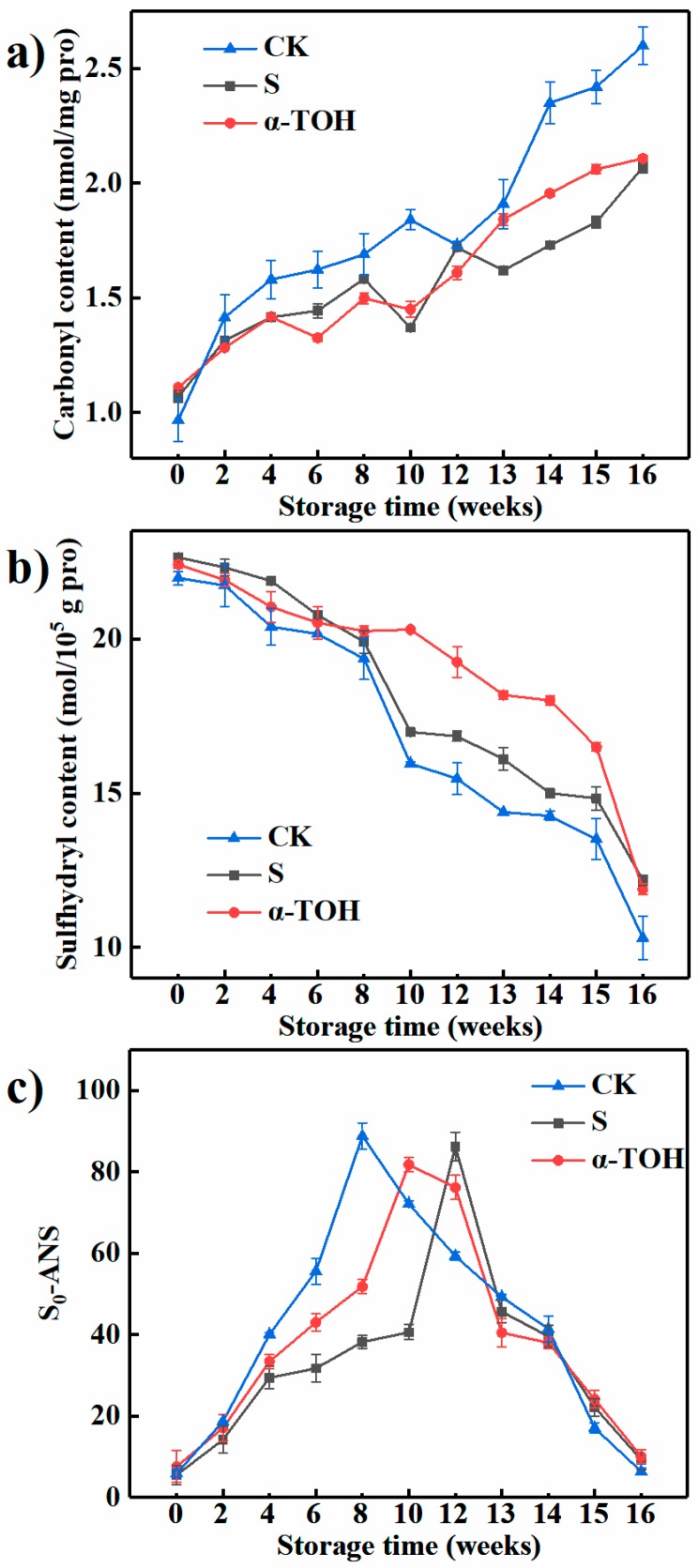
Effects of α-TOH on the carbonyl content (**a**), total sulfhydryl content (**b**) and surface hydrophobicity (**c**) of sturgeon surimi during 16-week frozen storage at −18 °C. Bars represent the standard deviation from triplicate determinations (*n* = 3; 3 means 3 replicates).

The formation of extra carbonyl groups on protein is one of the most important changes in protein oxidation [22]. Levine et al. [23] reported that the carbonyl content can be used as an indicator of protein oxidation. As shown in Figure 4a, after 16 weeks of frozen storage, the carbonyl contents of the CK group, S group and α-TOH group were 2.60 ± 0.082, 2.11 ± 0.013 and 2.11 ± 0.026 nmol/mg protein, which were respectively increased by 168.87%, 93.46% and 81.80% in comparison with their initial values. -NH or -NH_2_ groups on the proteins side chains are vulnerable to oxidation and thereby transformed into carbonyls [24]. The result showed that protein oxidation in sturgeon surimi during frozen storage caused the increases in carbonyl groups. After 16 week-frozen storage, there was no significant difference in carbonyl content between the S group and the α-TOH group (*p* > 0.05).

Fish myosin contains an amount of sulfhydryl groups, which easily form disulfide bonds by oxidation. Sulfhydryl groups play an important role in maintaining the three-dimensional structure of myosin [25]. As shown in Figure 4b, after 16 weeks of frozen storage, the sulfhydryl contents of the CK group, S group and α-TOH group were respectively reduced by 53.07%, 46.20% and 46.98% compared with their initial values, suggesting that α-TOH and cryoprotectant significantly prevented the formation of disulfide bonds and maintained the integrity of myosin structure. There was no significant difference in sulfhydryl contents between the S group and the α-TOH group after 16 weeks of frozen storage (*p* > 0.05).

Surface hydrophobicity (S_0_-ANS) represents the relative content of hydrophobic residues on the surface of protein and reflects the changes in protein conformation [26]. When frozen storage time increased between 0 and 12 weeks, the S_0_-ANS of all samples exhibited a significant elevation (Figure 4c). The hydrophobic residues were mostly embedded in myosin structure in fresh myosin and showed a lower S_0_-ANS. The increase in S_0_-ANS suggested that frozen storage extended myosin conformation, which exposed more hydrophobic amino acid residues to bind to the ANS fluorescent probe [27], resulting in an elevation in the S_0_-ANS of myosin. When frozen storage time was more than 12 weeks, the S_0_-ANS of myosin gradually decreased (Figure 4c). This could be ascribed to that, when hydrophobic groups raised to a certain level, myosin began to aggregate due to the enhanced hydrophobic interactions, and the hydrophobic groups were wrapped in aggregates, resulting in a decrease in S_0_-ANS [28]. After 16 weeks of frozen storage, the difference in S_0_-ANS between S group and α-TOH group was not significant (*p* > 0.05).

From these results, compared with the control, α-TOH and conventional cryoprotectant exhibited the retardation effect on carbonyl formation and sulfhydryl oxidation during frozen storage. In addition, these results also indicated that the protective effect of α-TOH on sturgeon surimi was similar to that of conventional cryoprotectant, which could protect proteins from oxidation. A previous report showed that α-TOH could retard protein oxidation in terms of carbonyl content and total sulfhydryl content [11].

### 2.4. Effects of α-TOH on the Gel Properties of Sturgeon Surimi during Frozen Storage

**Figure 5:**
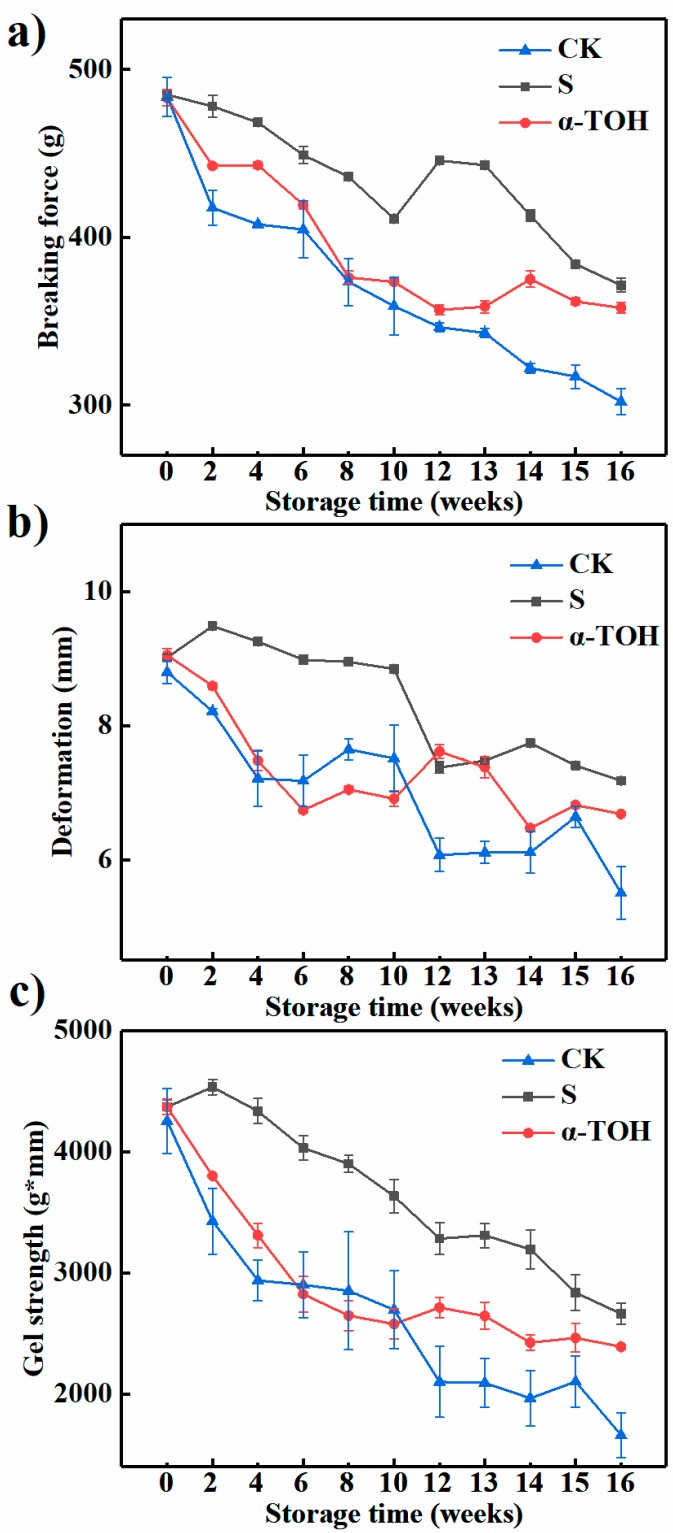
Effects of α-TOH on the breaking force (**a**), deformation (**b**) and gel strength (**c**) of sturgeon surimi during 16-week frozen storage at −18 °C. Bars represent the standard deviation from triplicate determinations (*n* = 3; 3 means 3 replicates).

Texture directly reflects the quality of frozen surimi [27]. As shown in Figure 5, the CK group had the lowest breaking force, deformation and gel strength (*p* < 0.05) throughout the storage period. After 16 weeks of frozen storage, breaking force, deformation and gel strength of the CK group were decreased by 37.56%, 37.45% and 60.95%, compared to their initial values. Additionally, breaking force, deformation and gel strength of S group were decreased by 23.44%, 20.41% and 39.06% after 16 weeks, while breaking force, deformation and gel strength of α-TOH group were respectively decreased by 25.88%, 26.17% and 45.28% compared with their initial values. This result showed that α-TOH significantly (*p* < 0.05) prevented the decrease in gel strength of surimi, and it could suppress the protein denaturation during frozen storage. When cryoprotectants were not incorporated, the rapid decrease in gel properties during frozen storage was observed in other fish muscle systems [29].

The decrease in gel-forming ability of fish muscles during frozen storage was relevant to the freezing denaturation of surimi actomyosin due to the aggregation of protein chains [30]. Denaturation or degradation of myosin during frozen storage resulted in an inferior gel network formation and a lower elasticity with poor water-holding capacity in the gel network [29]. In the present study, the decrease in gel properties was consistent with the decreases in protein content and sulfhydryl groups and the increases in carbonyl groups.

### 2.5. Effects of α-TOH on Water Holding Capacity (WHC) and Whiteness of Sturgeon Surimi during Frozen Storage

The changes in WHC reflect the variations in protein-water interactions and gel structure [31]. The decreases in the WHC were observed in the CK group, S group and α-TOH group during frozen storage (Table 1). After 16 weeks of frozen storage, the WHC of the CK group was decreased by 13.02%, whereas the S group and α-TOH group was respectively reduced by 6.46% and 7.19% compared to their initial values, indicating that less water was imbibed in the gel network of all samples. Denatured proteins induced by extended frozen storage possessed the low affinity for water. In addition, the formed gel matrix had the lower water holding capacity [32]. During 16-week frozen storage, WHC of the α-TOH group was lower than that of S group (*p* < 0.05). This result was consistent with the results of gel properties.

Whiteness affects the consumer acceptance of surimi-like products and is considered as a crucial quality indicator of surimi gels [31]. As shown in Table 2, after 16 weeks of frozen storage, the whiteness values of the CK group, S group and α-TOH group were respectively decreased by 7.37%, 5.28% and 2.94% compared with their initial values. The decrease of whiteness was probably caused by the abduction of pigment protein during the storage period, especially oxidized pigment to muscle proteins. Additionally, lipid oxidation in muscle might induce cross-linking of pigment proteins and muscle proteins through the free radical process during frozen storage [33]. During 16-week frozen storage, whiteness of the α-TOH group was higher than that of other groups (*p* < 0.05), which could be explained by the fact that α-TOH eliminated the free radicals generated during the automatic oxidation of fats and fatty acids [34]. Therefore, α-TOH group exhibited the higher whiteness.

### 2.6. Effects of α-TOH on the Microstructure of Sturgeon Surimi during Frozen Storage

**Figure 6:**
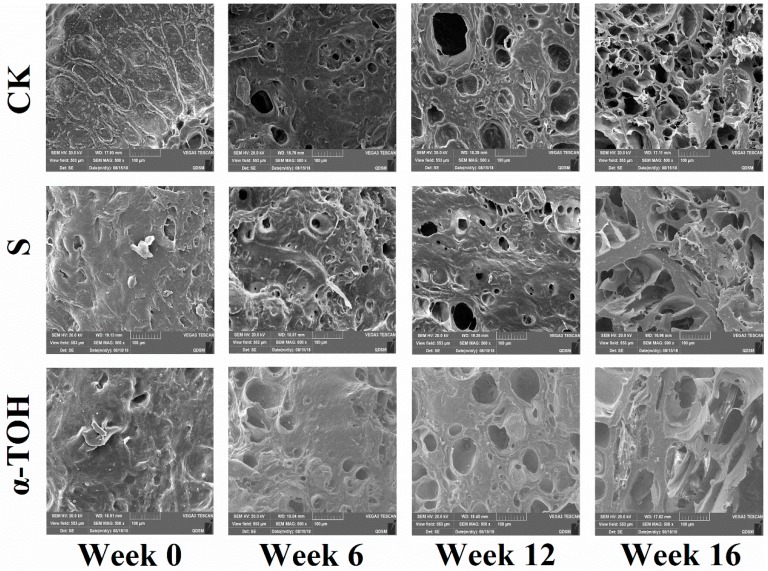
Effects of α-TOH on the scanning electron microscopy (SEM) images (500×) of sturgeon surimi during 16-week frozen storage at −18 °C.

The changes of microstructure of sturgeon surimi gel during frozen storage is shown in Figure 6. At week 0, the photos of all lyophilized sturgeon surimi samples displayed a regular dense structure with a well-organized three-dimensional network, and this allowed the surimi gels to maintain a certain elastic characteristic. Since week 6, three groups had a coarser and no-uniform network with the voids or cavities, suggesting that frozen storage resulted in the poor gel-forming ability of surimi proteins, which was consistent with the results of gel properties (Figure 5) and water-holding capacity (Table 1). This should be owing to the occurrence of more and more serious protein aggregation with the increase of frozen storage time [32]. After 16-week frozen storage, the gels of S and α-TOH group showed the nearly same microstructure and had the fewer cavities in comparision with that of the CK group, suggesting the prevented effect of the additives on protein denaturation. These results indicated that the protective effects of α-TOH on sturgeon surimi was comparable to those of the conventional cryoprotectant.

## 3. Materials and Methods

### 3.1. Materials and Reagents

Juvenile hybrid sturgeons (*Acipenser gueldenstaedti Brandt* ♀ × *Acipenser schrenckii Brandt* ♂) (about 1.5–2.0 kg) were obtained from an indoor cement pool at the Chengyang aquatic farm (Qingdao, Shandong, China). α-TOH was bought from Sigma-Aldrich Co. (Shanghai, China). Sorbitol and sucrose were purchased from Qingdao Jinbeiou Biological Co., Ltd. (Qingdao, Shandong, China). Other reagents used were of analytical grade and commercially available.

### 3.2. Preparation of Sturgeon Surimi

Sturgeons were transported into water according to a fish/water ratio of 1:10, (*w*/*v*) and carried to the laboratory in less than 1 h. Sturgeon surimi was prepared as per the method described by Zuraida et al. [35] with some modifications. After sturgeons were stunned, sturgeon meat was obtained by removing fins, skin, viscera and head. Next, sturgeon meat was washed with tap water at low temperature (5–10 °C), minced using a meat grinder, and rinsed according to the following procedure. Minced sturgeon meat was rinsed with five volumes of cold water once and 0.25% NaCl solution once. After dewatering, 8% of a conventional cryoprotectant (4% sorbitol and 4% sucrose) or 0.1% α-TOH was added into fresh surimi and mixed evenly. Surimi without cryoprotectant or α-TOH was used as the control group. The sturgeon surimi was packaged in a polyethylene bag and frozen at −18 ± 2 °C. The indexes were measured at 0, 2, 4, 6, 8, 10, 12, 13, 14, 15, and 16 weeks. Before the determination of all indexes, frozen sturgeon surimi was partially thawed at 4 °C overnight.

### 3.3. Microbiological Growth and pH Analysis

Sturgeon surimi (10 g) was added to a sterile homogeneous cup containing 90 mL of sterile 0.85% physiological saline before homogenization for 1 min. For microbial count analysis, a ten-fold dilution of sturgeon surimi homogenates were mixed with nutrient agar, followed by incubation at 37 °C for 48 h. For pH analysis, 10 g sturgeon surimi was homogenized thoroughly with 90 mL of distilled water.

### 3.4. Determination of Water-Extractable Protein (WEP) Content and Salt-Extractable Protein (SEP) Content

For the extract of WEP, sturgeon surimi (10 g) was homogenized with 10 mL deionized water for 2 min and then placed at 4 °C for 30 min before centrifugation for the supernatant. Then, 100 mL of Tris–HCl buffer (50 mM KCl, 20 mM Tris, pH 7.0) was added to the remained precipitate, and repeated the above extract procedure for supernatant. The mixture of these two supernatants were regarded as WEP. For the extract of SEP, 100 mL of Tris–HCl buffer (0.6 M KCl, 20 mM Tris, pH 7.0) was added to the remained precipitate and homogenized and placed at 4 °C for 60 min, and centrifuged at 10,000× *g* for 15 min at 4 °C using a GL-21M refrigerated centrifuge (Xiangyi Instrument Co. Ltd., Changsha, China). The obtained supernatant was regarded as SEP. The protein concentration of extracted supernatant was determined with the biuret method [36].

### 3.5. Determination of Protein Carbonyls, Protein Total Sulfhydryl and Surface Hydrophobicity

The protein carbonyl content was determined as per the method described by Soyer et al. [37]. The results are expressed as nmol carbonyls/mg protein.

The total sulfhydryl (SH) content was measured according to the method of Benjakul et al. [38] with minor modifications. 1 mL of myofibrillar protein from sturgeon surimi (2 mg/mL) was added into 9 mL of 0.2 M Tris–HCl buffer (pH 6.8, 8 M urea, 2% SDS, and 10 mM EDTA) and 4 mL of the mixture was added into 0.4 mL of a 0.1% 5,5′-dithiobis (2-nitrobenzoic acid) (DTNB) solution, followed by incubation at 40 °C for 25 min before measuring the absorbance at 412 nm using a microplate reader (Bio-Tek, 135 Winooski, VT, USA). The total SH content was evaluated with the extinction coefficient of 13,600 M^−1^ cm^−1^ and expressed as nmol/10^5^ mg protein. A blank was the sample with 0.1 M phosphate buffer.

Protein surface hydrophobicity was fluorometrically assayed according to the method of Benjakul et al. [38]. Myofibrillar protein from the sturgeon surimi were diluted to 0–1 mg/mL in 10 mM phosphate buffer (pH 6.0, 0.6 M KCl) and 10 μL (8 mM) 1-anilinonaphthalene-8-sulphonic acid (ANS) was added into the diluted protein solution (2 mL) for 10-min reaction in the dark. The fluorescence of ANS-conjugates at an excitation wavelength of 374 nm and an emission wavelength of 485 nm was measured on a HITACHII F-4600 spectrofluorometer (Hitachi High-Technoligies Corporation, Tokyo, Japan).

### 3.6. Sturgeon Surimi Gel Preparation

Partially thawed sturgeon surimi cubes (~3 cm) were chopped in a mixer (National Model SY-5, Guangzhou, China) for 2 min, followed by additional chop with 2.0% salt (*w*/*w*) for 2 min, After the final moisture content of the sample was adjusted to 80% with iced water [39], the resulting samples were stuffed into artificial casings of 3 cm diameter, sealed and then treated at 90 °C for 20 min. All the resulting gels were immediately cooled to room temperature in an iced water bath and stored at 4 °C overnight until further testing.

#### 3.6.1. Determination of Gel Properties

The breaking force (g) and the deformation (mm) were measured using a texture analyzer (TMS-Pro, Food Technology Co., Sterling, VA, USA) equipped with a spherical plunger of 5 mm diameter (60 mm/min depression speed). The gel strength (g × mm) is the product of the breaking force and deformation [40].

#### 3.6.2. Determination of Water-Holding Capacity and Whiteness

The water-holding capacity (WHC) was measured according to the method described by Himonides et al. [41] with some modifications. Chopped gels (about 1.5 g, W_1_) were wrapped in two-layer filter paper, put in the centrifuge tube, centrifuged at 3800× *g* for 15 min. After centrifugation, followed by weighting of the gels again (W_2_). The WHC is calculated as:

WHC (%) = [1 − (W_1_ − W_2_)/W_1_] × 100 (1)

Whiteness of surimi gels was evaluated by measuring the L* (lightness), a* (redness/greenness), and b* (yellowness/blueness) values using a WSC-S colorimeter (Shanghai Precise Instrument Co., Ltd. Shanghai, China). Whiteness is calculated as:

Whiteness = 100 − [(100 − L*)^2^ + a*^2^ + b*^2^] ^1/2^ (2)

#### 3.6.3. Microstructure of Surimi Gels

For scanning electron microscopy (SEM) observation, the lyophilized surimi gels (2–3 mm thick) were mounted on a bronze stub and sputter-coated with gold, followed by observation with SEM (JSM-5800 LV, JEOL, Tokyo, Japan) at an acceleration voltage of 20 kV.

### 3.7. Statistical Analysis

One-way analysis of variance (ANOVA) and Duncan’s multiple range tests were performed in SPSS 19.0 software (IBM, Armonk, NY, USA) to determine significant differences between variables.

## 4. Conclusions

After 16-week frozen storage, the CK, S and α-TOH group displayed the decrease in WEP, SEP, total sulfhydryl, gel strength, WHC and whiteness, and the increase in total viable count, pH values and carbonyls contents, suggesting that conventional cryoprotectant and α-TOH effectively retarded the changes of these indices. In terms of protein contents, protein carbonyls, protein total sulfhydryl and surface hydrophobicity, α-TOH showed comparable effect to the conventional cryoprotectant. The gel properties and WHC of α-TOH group were worse than S group during 16-week frozen storage, however, α-TOH group exhibited the lower TVC and pH and the higher whiteness compared to the S group. Therefore, α-TOH is a promising substitute for conventional cryoprotectant in maintaining the quality of sturgeon surimi gel during frozen storage. The results of this study showed that α-TOH exhibited high potency in retarding the changes of sturgeon surimi occurring during frozen storage, suggesting great potential for its utilization in surimi products.

## Figures and Tables

**Table 1:** Effect of α-TOH on the water-holding capacity of sturgeon surimi during frozen storage at −18 °C for 16 weeks.

Storage Time (week)	Water Holding Capacity (%)		
	CK	S	α-TOH
0	84.51 ± 0.006 ^a,A^	85.11 ± 0.002 ^a,A^	84.80 ± 0.030 ^a,A^
2	83.21 ± 0.005 ^a,b,B^	84.90 ± 0.007 ^a,A^	84.46 ± 0.003 ^a,A^
4	82.23 ± 0.023 ^a,b,c,B^	84.61 ± 0.010 ^a,A^	84.05 ± 0.008 ^a,A^
6	80.52 ± 0.018 ^b,c,d,B^	84.57 ± 0.011 ^a,A^	83.51 ± 0.018 ^b,A^
8	80.44 ± 0.009 ^c,d,C^	84.33 ± 0.004 ^a,A^	82.93 ± 0.021 ^b,c,B^
10	79.06 ± 0.000 ^d,e,C^	83.64 ± 0.010 ^a,A^	81.76 ± 0.013 ^b,c,B^
12	78.60 ± 0.008 ^d,e,B^	81.97 ± 0.022 ^a,b,A^	81.53 ± 0.012 ^b,c,A^
13	78.48 ± 0.015 ^d,e,C^	81.70 ± 0.014 ^b,c,A^	80.44 ± 0.019 ^b,c,B^
14	77.32 ± 0.011 ^e,f,C^	81.10 ± 0.053 ^b,c,A^	79.51 ± 0.004 ^b,c,d,B^
15	76.71 ± 0.010 ^e,f,C^	80.11 ± 0.008 ^c,d,A^	78.90 ± 0.016 ^c,d,B^
16	73.54 ± 0.008 ^f,C^	79.64 ± 0.003 ^d,A^	78.75 ± 0.017 ^d,B^

Data were expressed as means ± standard deviations (*n* = 3). Mean values with different letters within rows (^a–f^) or columns (^A–C^) are significantly different at *p* < 0.05.

**Table 2:** Effect of α-TOH on the whiteness of sturgeon surimi during frozen storage at −18 °C for 16 weeks.

Storage Time (week)	Whiteness		
	CK	S	α-TOH
0	72.75 ± 0.324 ^a,A^	72.67 ± 0.357 ^a,A^	73.09 ± 0.605 ^a,A^
2	71.16 ± 0.308 ^a,b,B^	72.55 ± 0.871 ^a,A^	72.78 ± 1.162 ^a,b,A^
4	71.18 ± 0.543 ^a,b,c,B^	71.72 ± 0.478 ^a,B^	72.42 ± 1.400 ^a,b,c,A^
6	71.02 ± 0.609 ^a,b,c,d,B^	71.82 ± 0.672 ^a,A^	72.40 ± 0.290 ^a,b,c,A^
8	70.73 ± 0.322 ^a,b,c,d,C^	71.66 ± 0.255 ^a,B^	72.23 ± 0.020 ^a,b,c,A^
10	70.52 ± 0.485 ^a,b,c,d,C^	71.61 ± 1.258 ^a,B^	72.09 ± 0.777 ^a,b,c,A^
12	70.26 ± 1.099 ^b,c,d,B^	71.21 ± 0.476 ^a,b,A^	71.92 ± 0.900 ^a,b,c,A^
13	70.02 ± 0.156 ^c,d,B^	71.38 ± 0.715 ^a,b,A^	71.87 ± 0.520 ^a,b,c,A^
14	69.76 ± 0.358 ^c,d,C^	70.83 ± 0.556 ^a,b,B^	71.72 ± 0.541 ^a,b,c,A^
15	69.61 ± 0.747 ^d,B^	69.78 ± 0.425 ^b,c,B^	71.27 ± 1.875 ^b,c,A^
16	67.39 ± 1.049 ^e,C^	68.83 ± 0.932 ^c,B^	70.94 ± 0.113 ^c,A^

Data were expressed as means ± standard deviations (*n* = 3). Mean values with different letters within rows (^a–e^) or columns (^A–C^) are significantly different at *p* < 0.05.
